# Vision of Life’s Code: Molecular Probe Nanoarchitectonics for Deep RNA/DNA Illumination

**DOI:** 10.1021/acsnano.5c22282

**Published:** 2026-02-19

**Authors:** Linawati Sutrisno, Kewei Sun, Katsuhiko Ariga

**Affiliations:** † International Center for Young Scientists (ICYS), 52747 National Institute for Materials Science, 1-1 Namiki, Tsukuba, Ibaraki 305-0044, Japan; ‡ Vacuum Interconnected Nanotech Workstation, Suzhou Institute of Nano-Tech and Nano-Bionics, Chinese Academy of Sciences, Suzhou 215123, China; § Research Center for Materials Nanoarchitectonics (MANA), National Institute for Materials Science, 1-1 Namiki, Tsukuba, Ibaraki 305-0044, Japan; ∥ Graduate School of Frontier Sciences, The University of Tokyo, 5-1-5 Kashiwa-no-ha, Kashiwa 277-8561, Japan

**Keywords:** RNA, DNA, bioimaging, chemical probes, multiplex, in vitro, detection, biologist, chemist

## Abstract

Visualization of RNA and DNA provides a window of opportunity for the early detection of multiple diseases and approaches to manipulate them before any pathological processes occur. However, current imaging systems often fail to meet these demands due to the need for multiple RNA and DNA chemical probes, which increases experimental complexity, reduces awareness of false positives, and limits imaging accuracy in complex biosystems. In this work, we identify the key challenges associated with using multiple probes for RNA and DNA imaging and analyze the mechanisms and performance of existing imaging tools. This article also introduces a conceptual framework and proposes a multidisciplinary framework to guide the development of next-generation imaging technologies. Furthermore, we highlight how nanoarchitectonics-based molecular design can enable single-step multiplexed RNA–DNA imaging. Our goal is to provide valuable resources for both biologists and probe developers for choosing suitable molecular probes and further advancing their design, from initial concepts to commercial products—all from a biologist’s perspective.

## Graphical abstract

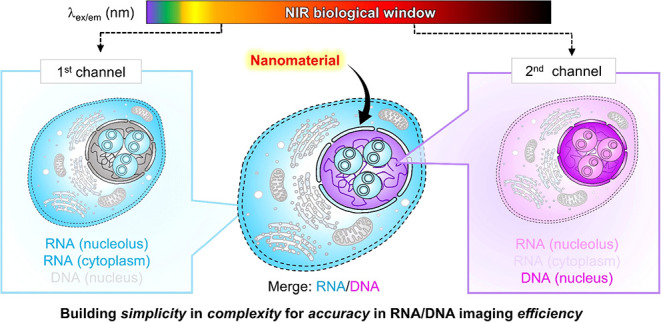

## Prologue

### General Background: Journey to Real Use of NANO

Human civilization has developed alongside usable functional materials, tools, and systems made from them. As society becomes more globalized, scientific discoveries and engineering inventions have the power to transform the quality of life for all of humanity. This became particularly evident during the Industrial Revolution and the subsequent development of science and technology over the past century. Advances in producing materials that perform specific functions and in processing those materials into precise structures that utilize their functions will create a more prosperous society.^1,2^ This perspective is being applied to the development of materials that address challenges in energy,^3–6^ the environment,^7–10^ and medicine.^11–14^

Two major developments have contributed to the advancement of functional materials since the 20th century. The first is the advancement of various chemical fields. The development and systematization of organic chemistry,^15–17^ inorganic chemistry,^18–20^ polymer chemistry,^21–23^ coordination chemistry,^24–26^ supramolecular chemistry,^27–29^ materials chemistry,^30–32^ and biochemistry^33–35^ have created methodologies for the systematic production of many substances. The other development is nanotechnology, which is supported by physics and related technologies. Following the development of materials chemistry, nanotechnology emerged in the mid-20th century and has advanced significantly since then.^36,37^ Nanotechnology enables the observation^38–40^ and manipulation^41–43^ of structures at the atomic and molecular levels, revealing new phenomena in the nanoscale region.^44–46^ This progress has significantly improved our scientific understanding of nanoscale regions. Consequently, nanotechnology has become an attractive area of research.

The goal of science and technology is to contribute to human and societal development. Translating the scientific knowledge of the nanoregion described above into useful materials is essential.^47–49^ Historically, we must promote a concept that integrates advances in materials chemistry with nanotechnology. One promising candidate is nanoarchitectonics, a postnanotechnology concept.^50^ It integrates materials chemistry and nanotechnology to construct functional materials from nanounits, such as atoms, molecules, and nanomaterials.^51,52^ This methodology is highly general and independent of the type of material or its application.^53,54^ Consequently, the concepts of materials nanoarchitectonics^55,56^ and molecular nanoarchitectonics^57,58^ have emerged.

It is important that these technologies lead to the development of human society and improvements in the convenience of daily life. This concept is deeply rooted among scientists. Although the concept of nanoarchitectonics is not explicitly stated, it is widely known that controlling nanostructures is necessary for a variety of applications. However, it cannot be said that research demonstrating functional improvements is scarce; for example, research discussing the usefulness from the user’s perspective may be lacking. Conversely, addressing these issues may clarify the importance of molecular nanoarchitectonics.

The main goal of this Review is to develop practical molecular nanoarchitectonics. As a target example, we would like to focus on biological applications, specifically simultaneous techniques for RNA/DNA imaging. Remarkable advances have been made in bioscience and biotechnology in the field of basic sciences. Medical technologies based on these advances have also made significant progress. In many cases, nucleic acids, such as DNA and RNA, are fundamental to these biological phenomena and technologies.^59–61^ While these targets are fundamental, it is interesting to consider whether useful technologies have been developed from the user’s perspective. Have molecular materials and technologies been developed that can simultaneously distinguish between DNA and RNA, image them precisely in real time, and closely monitor their behaviors? If not, what further developments are needed? What contributions can molecular nanoarchitectonics offer? These are the questions addressed in this review. The detailed objectives and methodology are described in the following sections.

### Focused Target: Identifying Critical Gaps in Intracellular RNA/DNA Imaging

Precise RNA/DNA imaging of complex biological systems requires a solid biological background for precise analysis, high requirements for molecular probes, and accurate bioimaging systems. However, in reality, most imaging systems fail to meet biological research demands due to the need for multiple RNA and DNA probes, which might increase complexity, low accuracy in biological systems, and a lack of awareness of false positives. Such problems lead to time-consuming experiments or complex analyses to collect reliable results, eventually leaving many RNA/DNA-related biological phenomena unnoticed for decades. Unlike conventional review articles, which are intended only to collect all existing references on popular topics, this review provides an overview of the untapped areas in the bioimaging field, a list of currently overlooked intracellular imaging problems, and an analysis from a biological perspective, inspiring researchers from different fields to explore new strategies relevant to their questions of interest.

This review is also intended for general readers and has the following objectives: (i) draw attention to long-standing, yet often ignored problems in applying multiple RNA and DNA probes for cellular imaging, (ii) provide an overview and comparison of current techniques for simultaneous RNA and DNA imaging, (iii) examine their underlying mechanisms to inform strategies for designing next-generation imaging tools, (iv) highlight our recent discovery that might have the potential to solve current problems, and (v) discuss new concepts and provide guidelines along with their limitations and possibilities to improve the development of RNA/DNA probes for in-depth biological studies.

### Previous Reviews on RNA and DNA Imaging Probes

Although many reviews have explored strategies for designing RNA or DNA probes,^62–66^ most have been written from the maker’s perspective (chemists) rather than the user’s (biologists), which has caused many synthesized probes to fail to reach practical use. In this section, we present relevant representative examples of existing reviews and identify several important points that have been consistently overlooked in previous reviews and current studies, which distinguish our review from others.

The review article by Baghdasaryan and Dai discussed the NIR-II photoluminescence properties of gold molecular clusters for preclinical *in vivo* NIR-II imaging.^62^ The review summarized the importance of NIR wavelengths for different types of organs, including the vasculature, brain, kidney, liver, and gastrointestinal organs, as well as for molecularly targeted tumor imaging and theranostic treatments. It also discussed the design, synthesis, and basic mechanisms of current NIR-II probes for targeting specific biological targets. The review also pointed out the need to consider controllable pharmacokinetics and the biodistribution of clusters to avoid long-term toxicity and highlighted several nanoparticles that have successfully reached preclinical and clinical studies, such as gold nanoclusters (Au NCs). Although gold nanoclusters have a high potential for biomedical imaging, they still face a major challenge in the synthesis with high yield. Unlike larger gold nanoparticles, whose properties are size-dependent but not atomically defined, Au NCs require precise atomic-level control to ensure a uniform electronic structure, optical properties, and stability. One important point that this review and much of the current research often overlook is that NIR imaging is widely used for *in vivo* studies but is still rarely applied in *in vitro* studies.

The review article by Le, Ahmed, and Yeo systematically summarizes recent advances in RNA imaging in both fixed and live cells.^63^ These advances include fluorescence in situ hybridization (FISH), single-molecule FISH (smFISH), rolling-circle amplification (RCA)-FISH, MERFISH, seqFISH, in situ sequencing (ISS), hybridization-based ISS, genetically encoded probes, such as CRISPR-associated (Cas) proteins for DNA targeting, the combination of fluorescently tagged RNA polymerase II with MS2 labeling of nascent mRNA to measure elongation rates, and multiple chemically synthesized probes for imaging endogenous RNA in living systems, such as 2′-O-methyl ribonucleotides and phosphorothioate backbones. The review also highlighted several biological insights that biologists can gain through RNA imaging, including an understanding of RNA throughout its functional life cycle, such as transcription, splicing, localization, translation, and degradation. Last but not least, the review also emphasized the need to integrate high-throughput methods with large-scale RBP–RNA interaction mapping approaches to capture the multidimensionality of RNA processing. Although this review focuses on the importance of RNA imaging, it overlooks the significance of simultaneous RNA and DNA imaging, which is essential for achieving more accurate imaging and gaining deeper biological insights.

The review article by Dong and co-workers highlighted the critical role of intracellular biomarker analysis for accurate disease diagnosis.^64^ The review summarizes recent advances in DNA biosensors that utilize programmable DNA sequences as molecular probes to monitor biomarkers. It outlined the fundamental structural components of DNA biosensors and their signal output mechanisms and discussed strategies for cellular internalization, including coincubation, nanocarrier-based delivery, and nanoelectroporation. The review also categorized the recent usage of DNA biosensors for detecting small molecules, RNAs, and proteins, as well as delivery methods. In addition, the review noted that live-cell analysis offers the advantage of enabling the long-term tracking of specific biomarkers over time. However, current DNA biosensors face significant limitations due to their susceptibility to degradation by intracellular nucleases, which often restrict them to single-use detection events, which causes difficulty in sustained intracellular monitoring. The review suggested that addressing this limitation through the development of innovative delivery strategies could enable the nondestructive delivery of DNA biosensors, thereby paving the way for continuous biomarker monitoring.

To date, most reviews have focused on DNA or RNA imaging, overlooking the need for RNA/DNA multiplex imaging, as well as the need for simple methods that are accessible to a wide range of researchers, including nonspecialists in the biological field.^62–65^ To our knowledge, no existing review has identified the importance of simultaneous RNA/DNA imaging for *in vitro* studies or offered a systematic analysis of the technologies capable of achieving it. This gap distinguishes our review from others. Beyond summarizing existing imaging tools, we also identify the conceptual barriers for the first time and introduce methodologies that may help overcome current RNA/DNA imaging limitations and accelerate the development of next-generation chemical probes in a more precise manner, aligned with the needs of real-world biological research. Our goal is to help biologists select the most suitable approaches for specific biological research, and activate future probe developers to design the next generation of RNA/DNA probes from conceptual ideas to commercial products from the biologists’ viewpoints.

## Problems, Properties, and Progresses

### Leap from Multistep to Single-Step Staining in a Complex Biosystem

Multiplex RNA/DNA imaging facilitates a powerful approach that opens up new avenues for studying the structural and functional dynamics of cellular activity. This method not only answers fundamental questions, such as “How do nucleus, nucleolus, and cytoplasm look in the cell?” and “How does RNA or DNA look in various cellular activities?”. More importantly, it is able to provide in-depth insights into RNA and DNA distribution as a result of the cell’s response to any biological processes. Simultaneous visualization of RNA and DNA allows researchers to study how RNA interacts with chromatin organization in the nucleus, shedding light on the molecular processes involved in cell growth, differentiation, and even response to external and internal stimuli at the single-cell level.

Combining DNA and RNA imaging probes within the same experiment is inherently problematic (Figure 1) because each probe is tailored to different targets and requires different experimental conditions.^65–67^ DNA is typically double-stranded and tightly packed within the nucleus or mitochondria, although it may be distributed to other cellular regions and become single-stranded during certain biological processes. In contrast, RNA is predominantly single-stranded and structurally more flexible. Such intrinsic differences influence how DNA and RNA respond to chemical reactions; therefore, simultaneous RNA/DNA visualization under a single set of conditions without RNA/DNA perturbation is required. In complex biosystems, these challenges become particularly pronounced. Overlapping signals due to structural and chemical similarities between DNA and RNA, probe–probe interactions, and variable labeling efficiency can distort imaging results. As a result, distinguishing real RNA or DNA signals from artifacts often requires multiple controls and validations. Rather than providing deeper insights, combining separate DNA and RNA imaging protocols results in complicated analyses and reduces data reliability.

**Figure 1:**
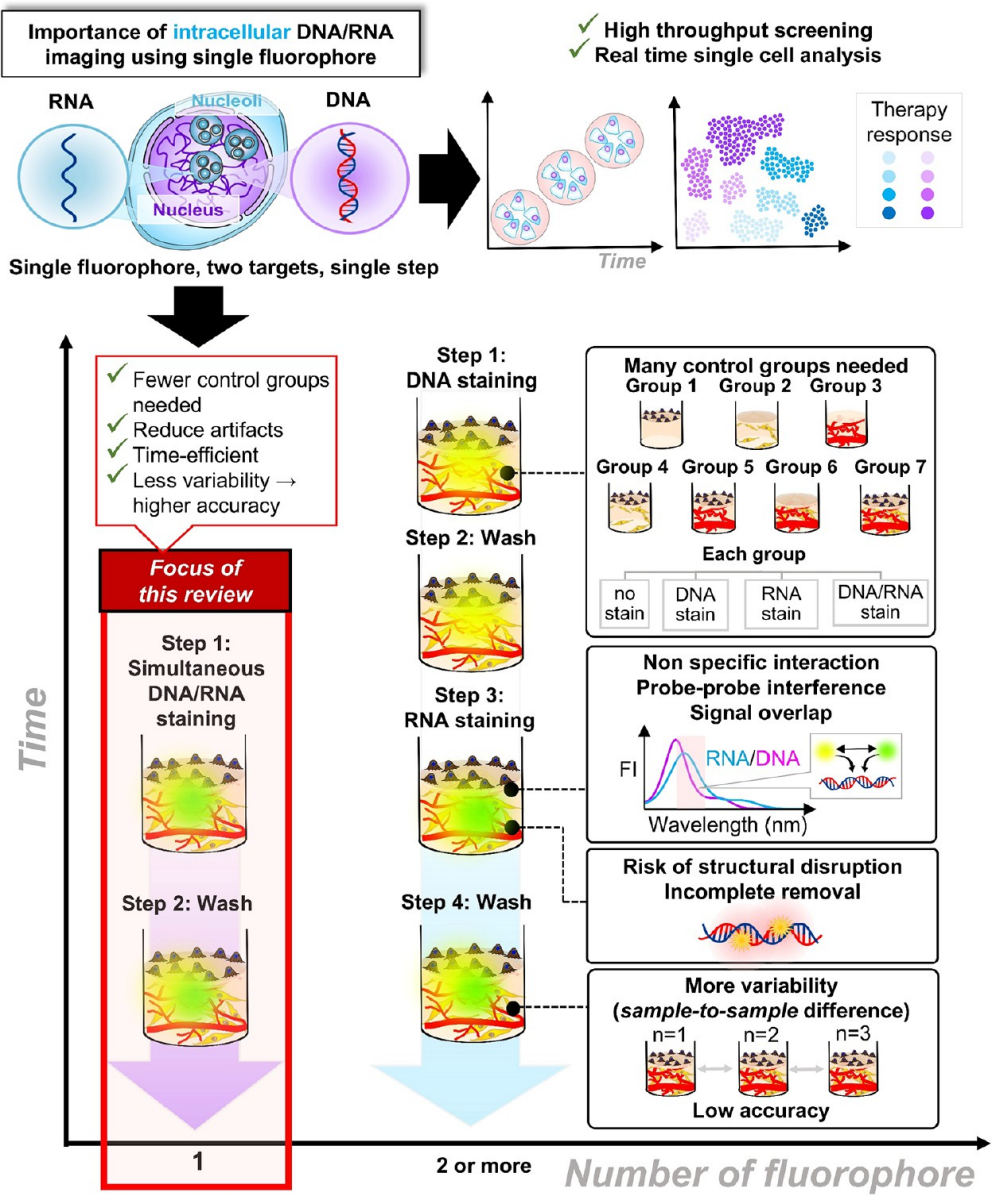
Building simplicity in complexity for high RNA/DNA imaging accuracy.

Considering the facts presented above, it is urgent to develop multiplex RNA–DNA cellular imaging that works under conditions compatible with both RNA and DNA in a single step. Such approaches have several advantages. First, they enable the direct observation of spatial and functional RNA/DNA relationships, providing a clearer and deeper understanding of how these interactions are regulated in real time, with minimal processing time, less variability, and fewer control groups. Second, multiplex imaging allows the simultaneous analysis of chromatin organization within individual cells, capturing dynamic molecular events that are often lost in bulk or sequential measurements while minimizing signal overlap from two different fluorophores. Third, by integrating both DNA and RNA information, researchers can better understand how structural genome features influence transcriptional heterogeneity across different cell types or states with minimal artifacts.

### Historical Flow: Transitioning from Difficult to Easy, yet Information-Rich RNA/DNA Imaging Techniques

Over the years, the field of nucleic acid imaging has evolved from early conventional approaches to more advanced techniques capable of simultaneously visualizing RNA and DNA (Figures 2 and 3a).^66,68–70^ To address phototoxicity and detect various RNA or DNA molecular conformations, Raman-based label-free methods for RNA and DNA have also been developed. However, they still suffer from overlapping vibrational spectra, the need for linear unmixing, as well as low resolution caused by weak signal intensity, which in turn leads to slow acquisition speeds during long-term imaging.^71^ FISH, while capable of codetecting multiple RNA or DNA targets that reach the genomic level,^72,73^ still suffers from the need for multiple rounds of hybridization, imaging, and alignment. The weak signal strength of FISH often necessitates signal-enhancement techniques,^74^ but these typically rely on high laser power or nonbiological window wavelength, thus causing phototoxic effects during prolonged imaging.^75^ Importantly, these complex methods require biological expertise and are therefore not suitable for researchers who are not specialists in the field.^76^ Overall, fluorescence imaging with a chemical probe is the simplest approach for labeling RNA and DNA and can be performed without specialized biological expertise. Although its high sensitivity allows researchers to easily detect changes in RNA or DNA within biological systems, both the molecular design and staining protocol must be well-optimized to obtain true-positive imaging results (Figure 3b).

**Figure 2:**
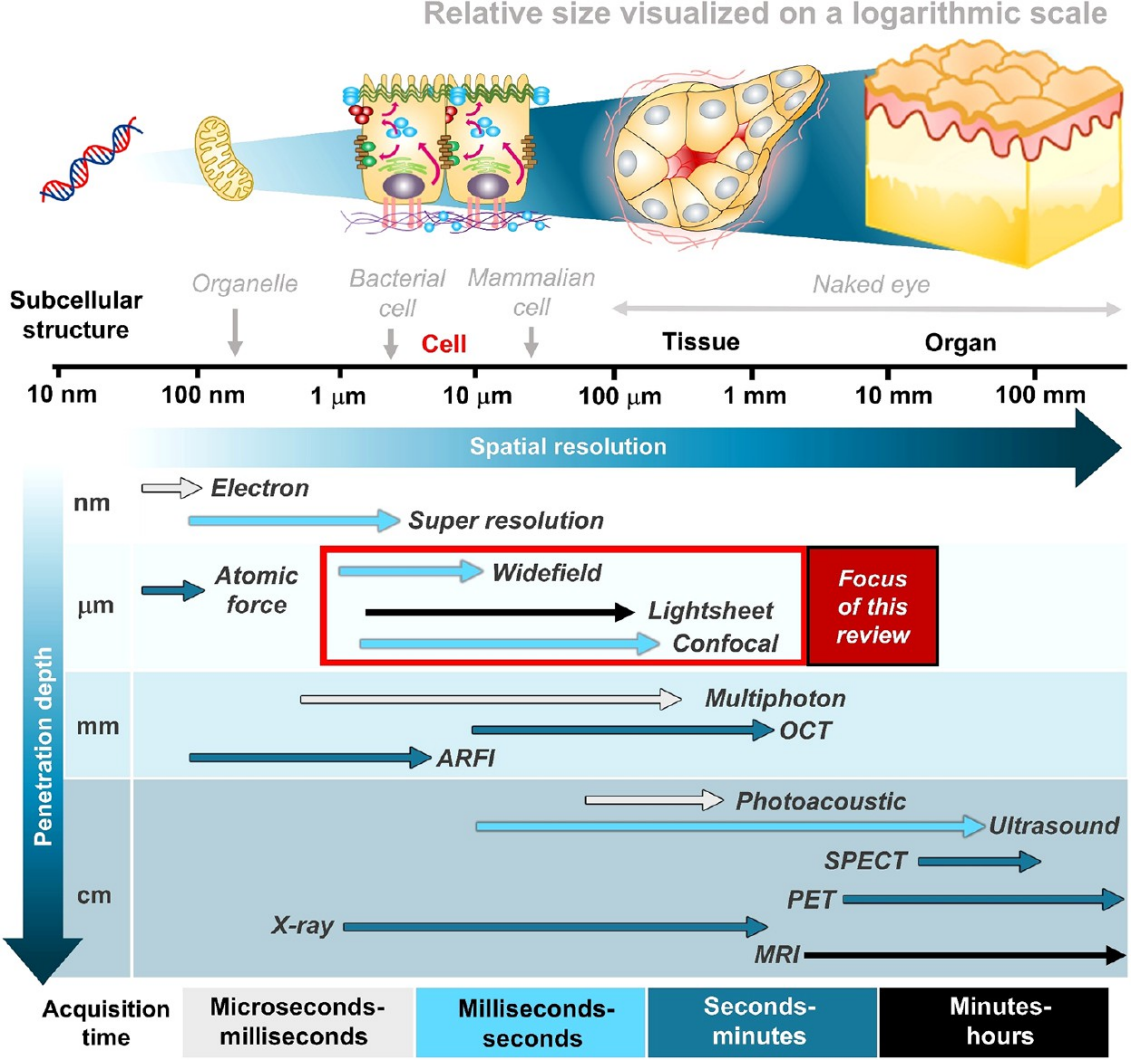
Overview of biomedical imaging technologies and the focus of this review.

**Figure 3:**
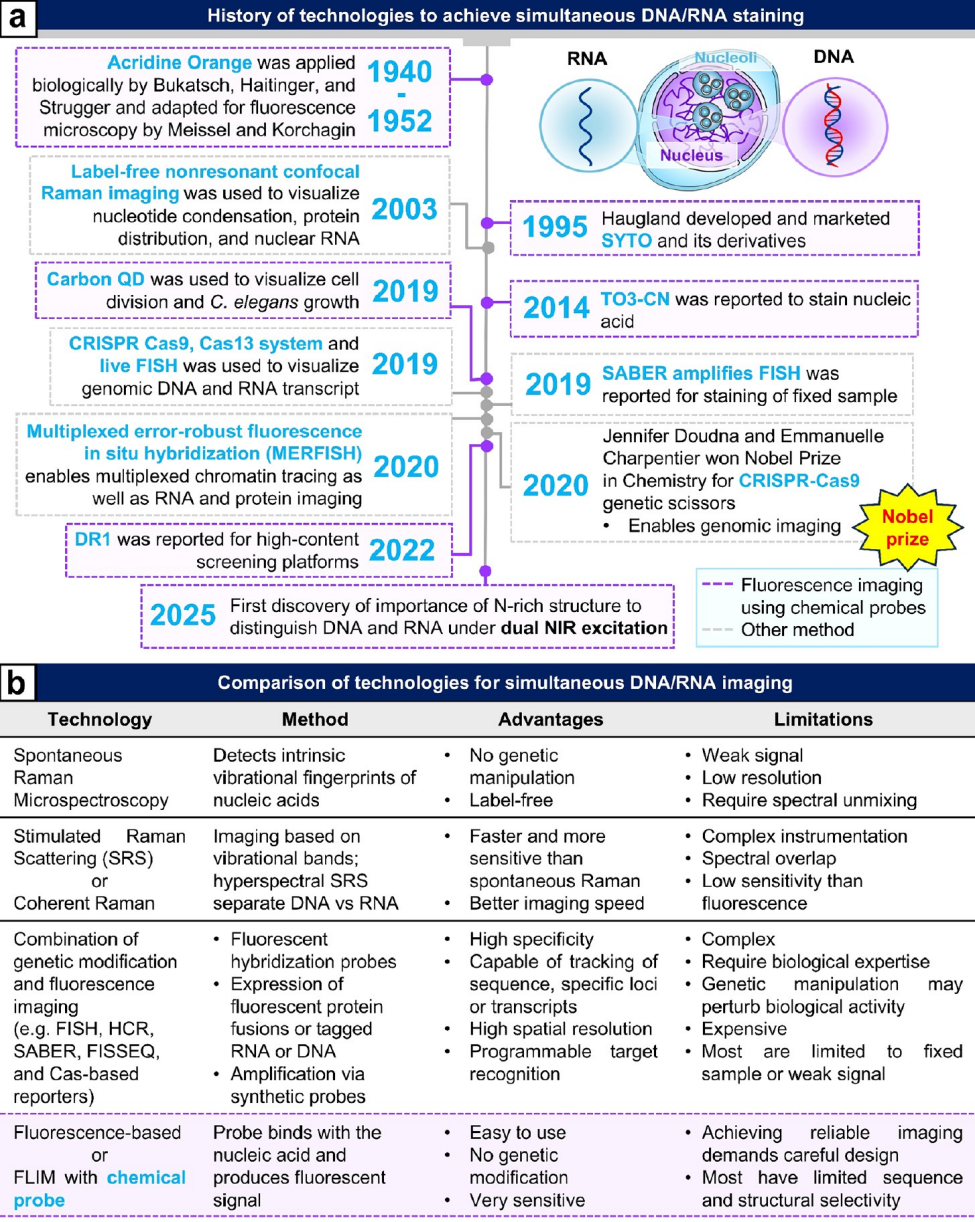
Overview of RNA/DNA imaging technologies. (a) Time sequence highlighting recent progress in simultaneous RNA/DNA imaging. (b) Comparative analysis of the existing methods for simultaneous RNA/DNA detection.

### Few Enough to Count on Our Fingers: Chemical Probes for Simultaneous Intracellular RNA/DNA Detection

Generally, chemical probes recognize and interact with DNA and RNA through physical and chemical interactions, enabling the specific visualization of nucleic acids in biological systems.^77^ Intercalating probes insert planar aromatic structures between stacked base pairs, stabilizing the nucleic acid–probe complex via π–π stacking. Groove-binding probes occupy the major or minor grooves of DNA, forming hydrogen bonds and van der Waals contacts, often providing sequence or structure selectivity. Many probes bind via electrostatic interactions with the negatively charged phosphate backbone, allowing nonspecific associations that facilitate imaging in certain contexts. Sequence-specific recognition is achieved by probes designed to form complementary base-pairing interactions with their targets, such as molecular beacons or peptide nucleic acids. Together, these mechanisms provide sensitive and reliable visualization of DNA and RNA, supporting the study of their localization, structural dynamics, and functional roles in both fixed and living cells.

Considerable progress has been made in the development of chemical probes for RNA and DNA imaging (Figures 3b and 4). However, it still faces complications due to imaging issues, such as low photostability, nonspecific binding, sensitivity to environmental factors (*e.g*., pH, ionic strength), and the most important challenge of simultaneously distinguishing RNA from DNA due to their similar structure. In the following sections, we provide recent advances in the development of chemical probes available for binding RNA and DNA, along with their respective advantages and limitations for cellular imaging, with special attention given to our recent discovery about the importance of nitrogen-containing core structures.

**Figure 4:**
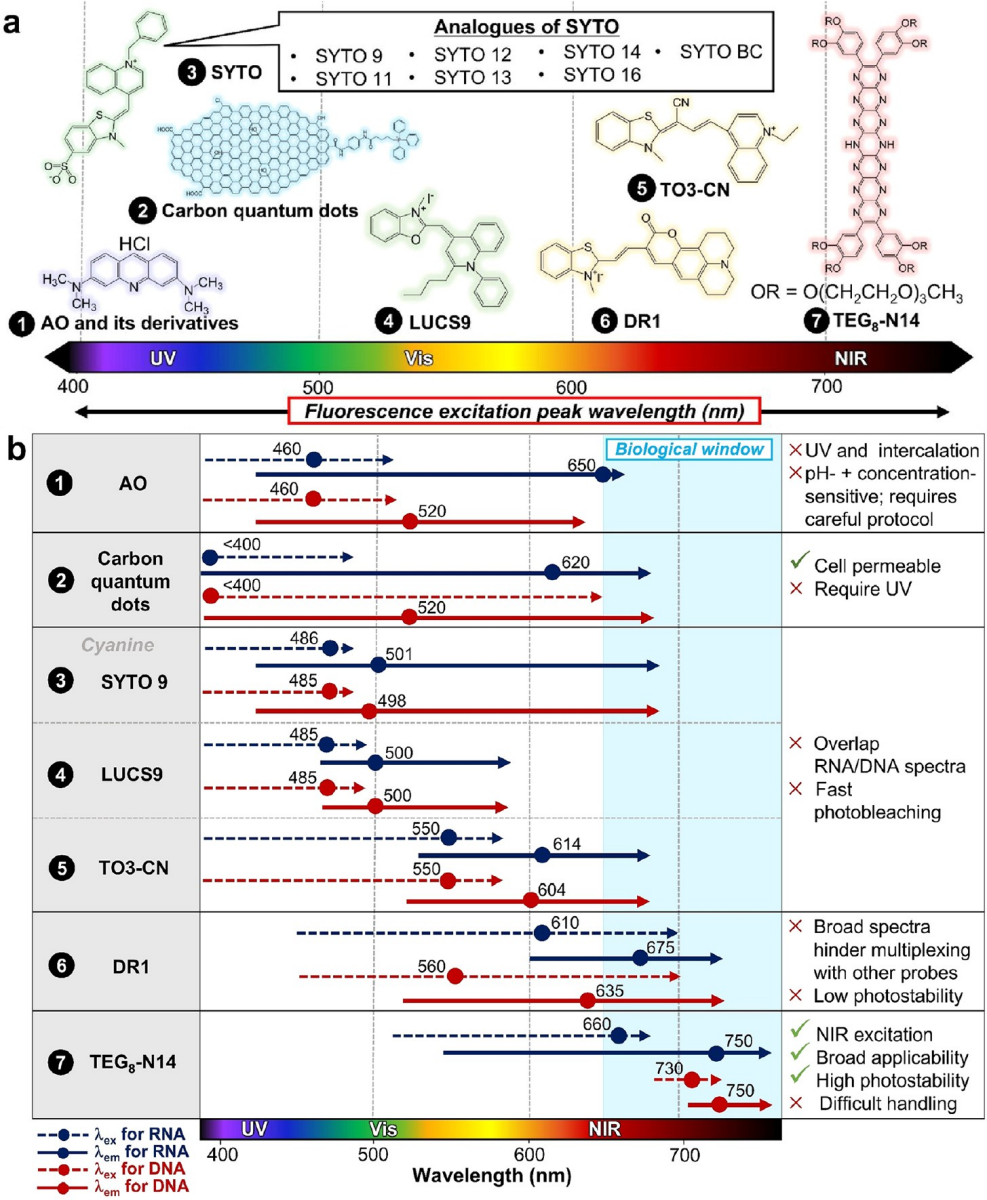
Selected examples of existing chemical probes for simultaneous RNA and DNA visualization. (a) Chemical structures of the existing compounds plotted according to their excitation wavelengths. (b) Comparison of current compounds for dual RNA/DNA imaging. The dots represent the peak excitation or emission wavelengths.

Early work largely relied on Acridine Orange (**AO**), which became a widely used tool due to its ability to selectively stain nucleic acids during 1940–1950.^78^ Several comprehensive reviews have documented these advances and the development of its derivatives,^79–81^ highlighting the optimized protocol,^82,83^ proposed possibility mechanisms of **AO** staining,^84,85^ and its diverse applications in bioimaging.^83,86,87^ **AO** consists of an acridine core formed by three fused aromatic rings with two dimethylamino side groups and can exist in multiple resonance-stabilized isomeric forms, including para-quinone, ortho-quinone, and an uncharged free-base configuration, enabling various interaction modes with biomolecules. In fixed and living cells, **AO** uptake and localization might be affected by the dynamic equilibrium between protonated and deprotonated species, which influences its intracellular distribution, aggregation state, and binding behavior. In biological environments, **AO** may exist as monomers or higher-order aggregates, with aggregation depending on the local concentrations in specific biological targets. In fixed cells, **AO** is proposed to bind predominantly to single-stranded RNA within the nucleolus and cytoplasm through external association and aggregate formation, which may be attributed to the structural flexibility of RNA. These binding states are reflected in the fluorescence behavior of **AO**, as microscopic visualization under 436 nm excitation reveals concentration-dependent emission changes corresponding to the monomeric and aggregated forms. Despite their utility, **AO** and its derivatives suffer from intercalation between base pairs, which causes an increase in the length of the DNA helix, distorts the steric molecular structure, and alters the spatial charge distribution.^88^ Additionally, their pH sensitivity and the requirement for UV excitation often cause unreliability in imaging;^89^ therefore, prestaining conditions require careful optimization to achieve accurate color in imaging. Although several acridine derivatives have been synthesized to increase membrane permeability and facilitate RNA or DNA binding,^90,91^ they still suffer from biased visualization due to their oversensitive properties (Figure 5a–c). Therefore, acridine and its derivatives are now more widely explored for theranostics applications rather than for long-term imaging (nowadays commercialized).^92^

**Figure 5:**
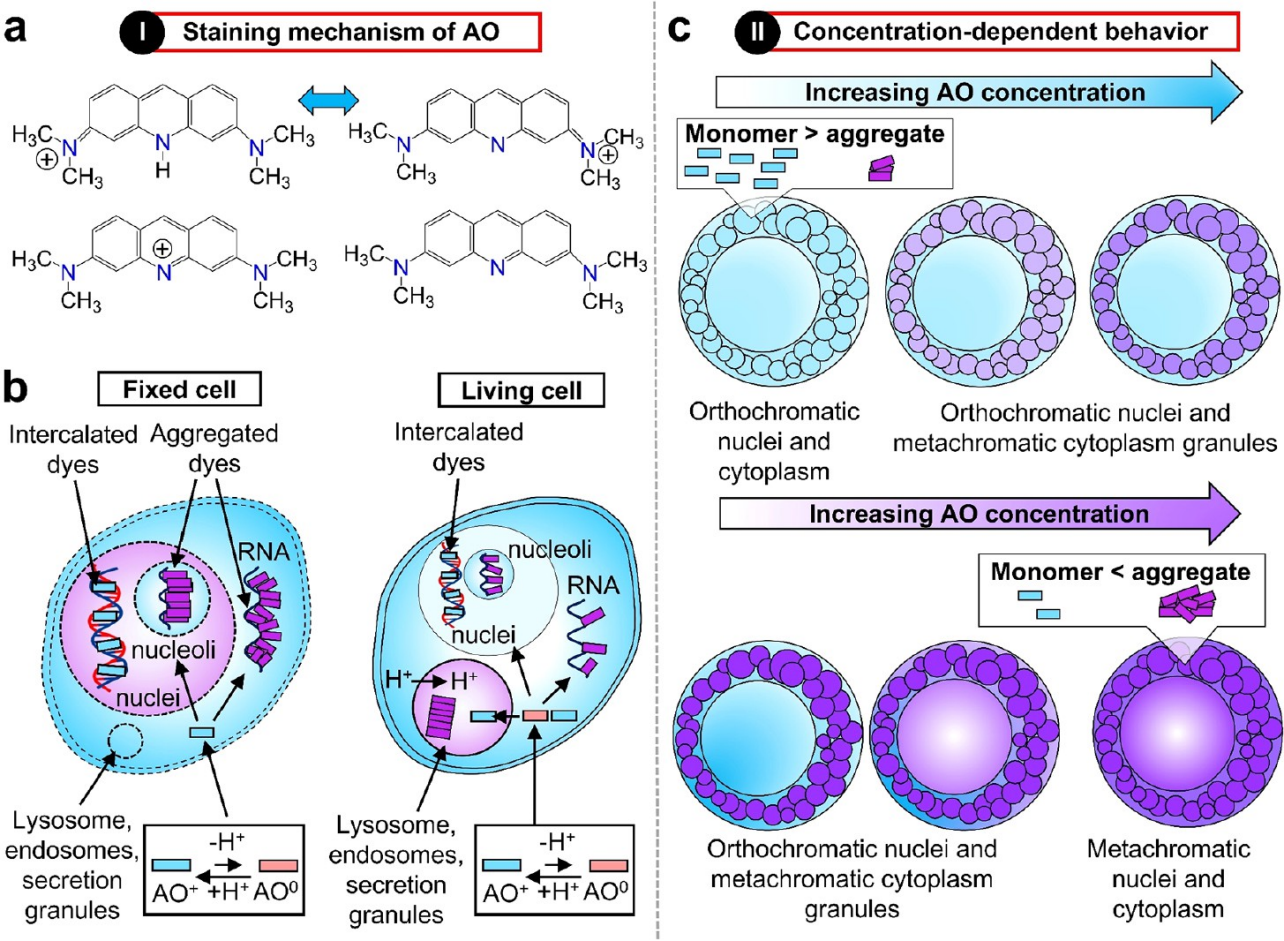
Structural, chemical, and fluorescence properties of Acridine Orange (AO). (a) Molecular structure and resonance forms of AO. The acridine core consists of three fused aromatic rings with two dimethylamino side groups. Resonance occurs among several possible isomeric forms, including para-quinone (top left and right), ortho-quinone (bottom left), and uncharged (unprotonated) free-base (bottom right) configurations. (b) AO behavior in fixed and live cells. Schematic representation of AO uptake and localization mechanisms, illustrating the equilibrium between protonated (cationic) and deprotonated (free base) species. AO monomers are shown as cyan bars, and AO aggregates appear as magenta bars. In fixed cells, AO binds to single-stranded RNA in the nucleolus and cytoplasm primarily through external aggregate association rather than intercalation, following the classical model. (c) Fluorescence behavior and microscopic visualization at varying AO concentrations under excitation at 436 nm.

To overcome the sensitivity problems of **AO**, cyanine-based dyes, denoted as **SYTO** dyes, and their derivatives were developed for RNA/DNA imaging (nowadays commercialized). Unlike **AO**, **SYTO** becomes highly fluorescent when bound to nucleic acids.^93,94^ **SYTO** can label both RNA and DNA in live or dead eukaryotic cells and Gram-positive bacteria. **SYTO** derivatives have been explored and are now commercially available in blue, green, orange, and red fluorescent variants based on their excitation and emission wavelengths. The **SYTO** family shares several features: ability to cross nearly all types of cell membranes, including those of bacteria and mammalian cells; high molar extinction coefficients greater than 50,000 cm^–1^ M^–1^ at their absorption maxima; low or almost no fluorescence when unbound (PLQY around 0.01); and strong fluorescence when bound to nucleic acids (PLQY > 0.4, increases nearly 40-fold). Although they share similar features, each **SYTO** derivative differs in properties such as cell permeability, the extent to which their fluorescence increases when bound to nucleic acids, along with their excitation and emission profiles, DNA versus RNA selectivity, and overall binding affinity. Due to their high molar absorption coefficients, they are compatible with a wide range of fluorescence-based instruments that use either laser excitation or traditional broadband light sources such as mercury- or xenon-arc lamps. Although **SYTO** binds to both RNA and DNA, its primary target and binding mechanism may vary with cell type and biological environment. Interestingly, in the use of nucleic acid dyes for staining for bacteria, RNA in certain species does not seem to bind **SYTO 13** effectively. Despite their advantages, **SYTO** dyes exhibit overlapping DNA/RNA spectra, rapid photobleaching (faster than **Hoechst 33342** and **DRAQ5**), limiting their suitability for prolonged or quantitative imaging that requires high-intensity laser illumination.

**LUCS-9** represents a refined **SYTO** family member and exhibits fluorescence upon binding to nucleic acids, with emission peaks at around 500 nm for DNA and 504 nm for RNA. Such slight spectral differences and low photostability also limit its capability for simultaneous imaging of both nucleic acid types. However, its cell-permeant ability makes it well-suited for both live and fixed-cell microscopy, as well as flow cytometry.

To overcome the photostability problem of cyanine-based dyes, Han and colleagues developed a highly photostable dye, cationic carbon quantum dots (**CQDs**), capable of distinguishing fluorescence upon binding to single-stranded RNA (ssRNA) and double-stranded DNA (dsDNA) in live cells.^67^ The cationic charge was introduced to the **CQDs** through the addition of polydopamine and PPh_3_^+^, enabling the nanodots to interact with both dsDNA and ssRNA via multiple noncovalent interactions, including ionic, π–π, and hydrogen bonding. Different interactions have been proposed for dsDNA and ssRNA: the high structural rigidity of dsDNA confines **CQDs** within the grooves, resulting in enhanced fluorescence from isolated particles, whereas flexible ssRNA acts as a **CQD** concentrator, bringing multiple **CQDs** into close proximity. Several attractive features make these **CQDs** promising for bioimaging, including compatibility with STED microscopy for high-resolution imaging, high photostability (higher than **Hoechst 33342**), and cell permeability, which have been demonstrated by their ability to monitor cell division and *C. elegans* growth. Despite their promising capabilities, the requirement for UV illumination restricts their application for prolonged imaging at short time intervals.

**TO3-CN**, a representative example of thiazole orange derivatives, was subsequently designed to improve the photostability of the previous development.^95^ Specifically, the introduction of a cyano (CN) group into the trimethine chain of the classical red-emitting **TO-3** dye enhances photostability, fine-tunes spectral properties, and modulates interactions with nucleic acids.^96^ **TO3-CN** exhibited several attractive features, including a large fluorescence Stokes shift (>40 nm), high quantum yield (>0.7), and low cytotoxicity. It showed enhanced brightness upon binding to DNA (approximately 7-fold increase, molar extinction coefficient more than 50,000 cm^–1^ M^–1^) and RNA (around 7-fold increase). However, the close spectral overlap of DNA and RNA limits its ability to discriminate between the two simultaneously. Therefore, under the same observation conditions (excitation 559 nm, emission 575–620 nm), fluorescence in MCF-7 cells was observed in both the nucleolus and nucleus.

**DR1**, a cyanine-based dye, provides a better strategy than **TO3-CN** for RNA/DNA spectral discrimination by binding DNA through both the major and minor grooves and RNA through electrostatic interactions.^97^ Although the distinct spectral signatures of DNA and RNA allow simultaneous discrimination, their low photostability is primarily due to the double-bond structure, which makes them highly prone to degradation during prolonged imaging. Given its potential phototoxicity, low photostability, and limitations for long-term imaging, **DR1** is better suited for high-content screening applications rather than real-time imaging.

To date, the majority of fluorescent probes used for intracellular imaging rely on UV–visible excitation, largely due to the limited sensitivity of conventional fluorescence microscopy systems in the near-infrared region. As a result, intracellular probes that operate efficiently under NIR excitation remain rare, and their successful implementation represents a significant technical breakthrough. In this context, we recently reported a significant advance that overcomes current probe limitations and microscopy constraints by developing an optimized staining strategy for N-rich structured compounds (denoted as **TEG**_**8**_ **-N14**) to simultaneously visualize RNA and DNA with dual near-infrared excitation (Figure 6a,b).^98^ Such NIR excitation not only minimizes phototoxicity and spectral crosstalk but also shows broad applicability for measuring multiple types of cell injury in different cell types. Docking simulations indicate that its binding to DNA is based on multiple interactions, starting with C–H···π interactions, groove binding, and anion−π interactions between the phosphate backbone and the electron-deficient extremities of its core unit. In contrast, for RNA, the interactions are more variable and depend on its secondary structure. One advantage of this probe is that it binds to DNA via groove binding rather than intercalation, which causes lower cytotoxicity and avoids distortion of the DNA double helix. Groove binding also reduces interference with DNA-binding proteins compared to intercalation, allows for higher binding selectivity when the staining protocol is properly optimized, and provides higher photostability compared to common intercalating dyes. Interestingly, its photostability is comparable to that of **SiR-DNA**, one of the most photostable probes developed to date. Due to its aggregation, the probe exhibits reduced cell permeability and low cellular brightness without the addition of a proper surfactant. Therefore, future development should focus on strategies to improve the cell permeability and cellular brightness and modify the chemical structure.

**Figure 6:**
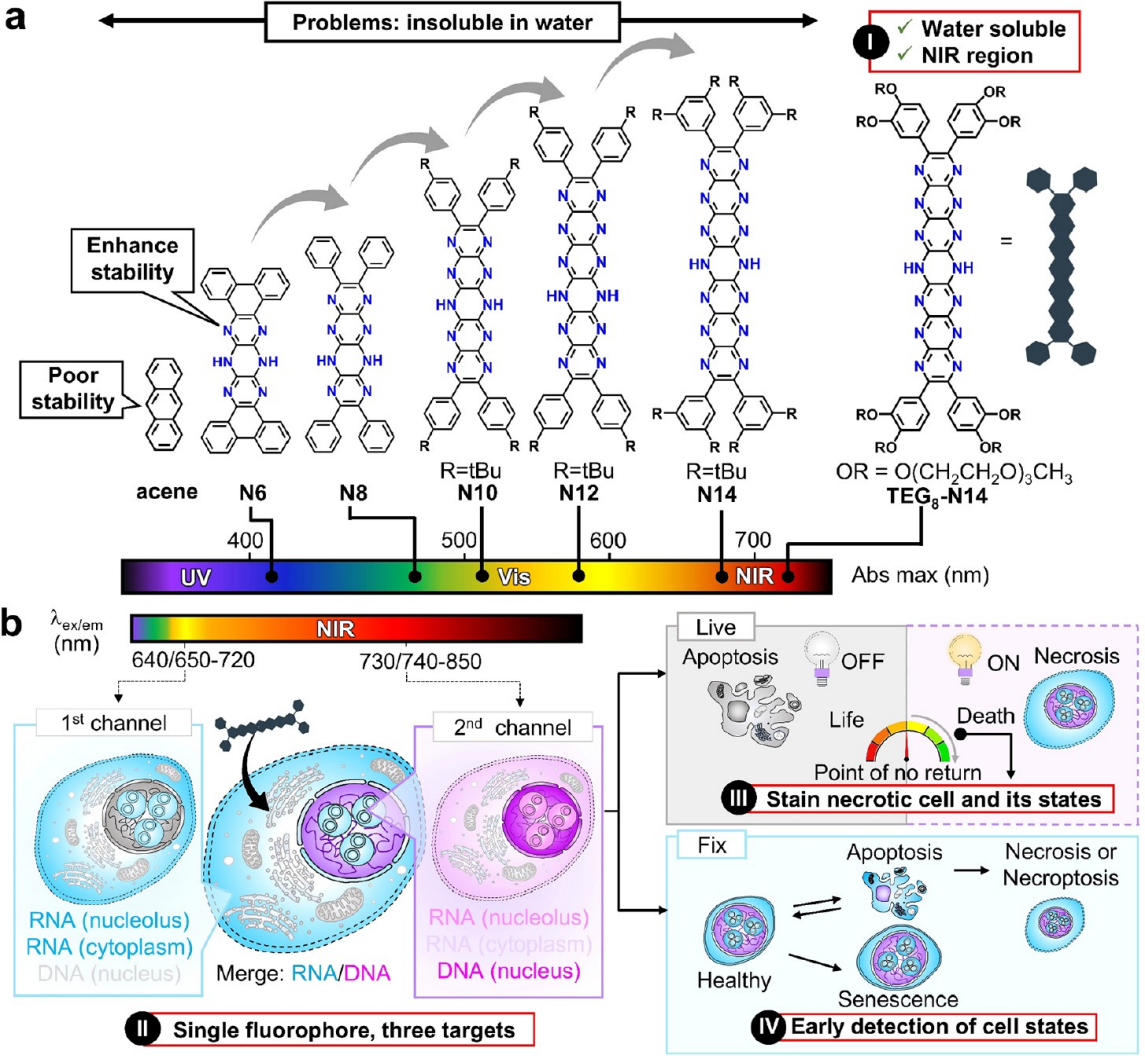
Historical development of pyrazinacenes: from molecular design to their first exploration in bioimaging. (a) Electronic absorption spectra of pyrazinacenes with an increasing number of fused pyrazine rings. (b) RNA–DNA and multiple cell-state discrimination using a single fluorophore (TEG_8_-N14) under NIR excitation.

In summary, although there are many probes that are able to bind DNA and RNA in cells, to date, only four types of probes—**AO** and its derivatives, **CQDs**, **DR1**, **TEG**_**8**_ **-N14**—have the capability for simultaneous DNA and RNA imaging. These four examples provide further possibilities as a fundamental framework for designing chemical probes with properties that extend beyond the simple combination of individual RNA or DNA probes. Specifically, such dual-function probes are essential for flow cytometric studies,^99^ high-throughput screening,^97^ histochemical observation in cells, bacteria, or viruses,^100^ observation of chromatin structural changes during repair pathways,^101^ cell cycle,^67^ or stress,^102^ which are crucial for advancing diagnostics and therapeutic developments.

## Promising Probes and Procedures

### What Kind of Fluorophore is the Best from a Biologist’s Perspective?

Although a perfect probe does not exist, critical design elements are essential for its optimal performance. In the following sections, we outline several key design criteria for developing chemical probes along with the limitations that significantly hinder their performance. We discuss several unusual concepts that have changed the traditional concept of chemical probe development. We also propose a way to adjust the specific application of probes, which is often overlooked by most researchers in the field.

### Biological Window: Gateway for Universal Application

The complex structure of biological cells and tissues often leads to opacity, primarily due to unwanted light scattering and absorption in the biological environment, which limits the penetration depth of optical imaging and causes unwanted autofluorescence from endogenous fluorophores.^103–105^ In many cells and tissues, the scattering coefficient exceeds the absorption coefficient by 10- to 1000-fold, making scattering the main factor that restricts both imaging depth and spatial resolution in conventional microscopy (Figure 2). Although various probes targeting nonbiological window regions have been developed,^106^ autofluorescence of specific cell types and phototoxicity during long-term imaging remain a concern, particularly when visualizing light-sensitive parts, such as DNA and RNA,^107^ which can potentially compromise the accuracy of the results. For cellular imaging applications, the focus of this review, the selection of an appropriate NIR biological window depends on the experimental context.^4,108,109^ For example, 3D cell cultures, such as spheroid, organoid, or *in vivo* imaging, require longer wavelengths for deep imaging compared to 2D systems.^110–113^ In addition, different cell types have different degrees of endogenous fluorophores, making it crucial to carefully choose fluorescence excitation and emission wavelengths tailored to each specific application.

### Photoluminescence Quantum Yield is Not the Primary Consideration

Although the photoluminescence quantum yield is an important photophysical parameter, a very high quantum yield is not necessarily the most critical factor, as probes may behave differently once localized to a specific area or interact with specific biomolecules in biological media.^114^ Some probes may show fluorescence enhancement after they interact with biomolecules. Therefore, cellular brightness after probe internalization should be given greater consideration.^115^

### Oversensitivity: A Source of Analytical Complexity

Although probe sensitivity is essential for sensing applications,^106,114,116^ multiple sensitivities complicate data interpretation and increase the requirement for rigorous calibration. For example, **AO** is sensitive to pH, aggregation, and protonation.^117^ Therefore, when detecting RNA changes in certain diseases with **AO**, many questions arise: “Are the observed signals due to RNA changes, aggregation changes, or pH differences?” or “Are there any nonspecific binding that causes a difference in fluorescence intensity?” Such excess responsive probes can amplify minor fluctuations, particularly in sensing applications, potentially leading to misleading fluorescence intensity quantification readouts unless carefully controlled and standardized. However, by using such probes, we can still accurately extract information about cell kinetics or morphological features. In summary, probes with just specific sensitivity are much preferred over those with high sensitivity.

### Trade-Off between Brightness and Photostability

By avoiding fast photobleaching, a probe can be used not only for quantitative and long-term imaging but also in super-resolution microscopy,^112,118^ which provides more detailed insights into biology-related processes. However, achieving a balance between brightness and photostability remains a major challenge in probe design, especially due to the aggregation-induced quenching effect or any unwanted interaction exhibited by the majority of molecular probes in biological media.^63,119,120^ Bright molecular probes often undergo rapid photobleaching under continuous illumination. In contrast, highly photostable fluorophores may have low brightness, reducing their detectability.^121^ To maximize a probe’s utility in long-term imaging and balance photostability and brightness over time, careful molecular design must be optimized to achieve both properties effectively.^122,123^ A good example of a molecular design that balances photostability and cellular brightness is pyrazinacene, which was developed by extending the number of fused pyrazine rings and introducing nitrogen atoms to enhance stability, as well as by TEGylation to improve its water solubility.^124^ Incorporating nitrogen atoms into an aromatic framework leads to tunable molecular orbital energies, resulting in an improved electron affinity and enhanced oxidative stability. For instance, pyridinic nitrogen atoms act as electron-withdrawing units, stabilizing azaaromatic systems relative to their parent hydrocarbon analogues. In particular, this stabilization is associated with a lowering of the frontier molecular orbital energies and depends not only on the number of nitrogen atoms introduced but also on their precise positions within the aromatic backbone. These positional effects influence the oxidation behavior, susceptibility to electrophilic attack, binding affinity, chemical interactions, and local electron density in biological environments, ultimately contributing to enhanced chemical stability.^125^ Although pyrazinacene has a self-quenching effect in aqueous media, its fluorescence “switch-on” properties upon interaction with RNA or DNA facilitate bioimaging applications while maintaining both photostability and brightness, allowing the monitoring of different types of cell injury (Figure 5a).

### Cell Permeability: Not Always an Obstacle to Bioimaging Applications

Cell permeability is a critical factor for tracking dynamic processes in living cells. Due to the highly specific nature of nuclear pores, it remains difficult for small molecular probes or other organic molecules to pass through and enter the nucleus. Therefore, tailoring the structure of chemical probes or adding a cell-penetrating agent must be considered for imaging biological events in the nucleus. However, the probe’s limited permeability does not necessarily preclude its practical application. For example, propidium iodide and DAPI have been widely employed to detect plasma membrane rupture and other forms of cell death.^125–128^ Therefore, even if a probe is impermeable, its specific application must be carefully tailored based on its properties. Additionally, such probes are still suitable for imaging fixed cells that require permeabilization. In the context of live-cell imaging, cell permeability is a primary criterion, but any potential changes during tracking must also be taken into account to ensure accurate and reliable imaging quantification (Figure 6b).

### Multiplexing: Mapping Organelle Interactions with Compatibility Across Probes

Multiplexed fluorescence imaging enables the simultaneous detection of multiple targets in a cell, revealing the relationship between targets in a single step.^114,129,130^ Without this capability, it is difficult to identify relationships among signals, analyze their interactions, and understand how these biological processes malfunction under pathological conditions. As a simple example, consider a case where signal A is high in a cell while signal B is low, and vice versa, perhaps because A suppresses B. Imaging A and B in separate cells would fail to reveal this relationship; only by measuring both signals simultaneously in the same cell with the same probes can this interaction be clearly observed.^131^ Another case, identifying changes in a single organelle, is not always sufficient to understand cell states. For example, in the late stage of necrosis, some cells become bright and smooth without RNA disruption in the nucleolus, while others do not. Similarly, during the senescence stage, observing only DNA is insufficient for the early detection of metabolic disruption (Figure 7).^98^ Therefore, RNA and DNA signals should be detected simultaneously and, if possible, multiplex probes should be used without interference of their compatibility to better understand the cell state in an easier and more precise way.

**Figure 7:**
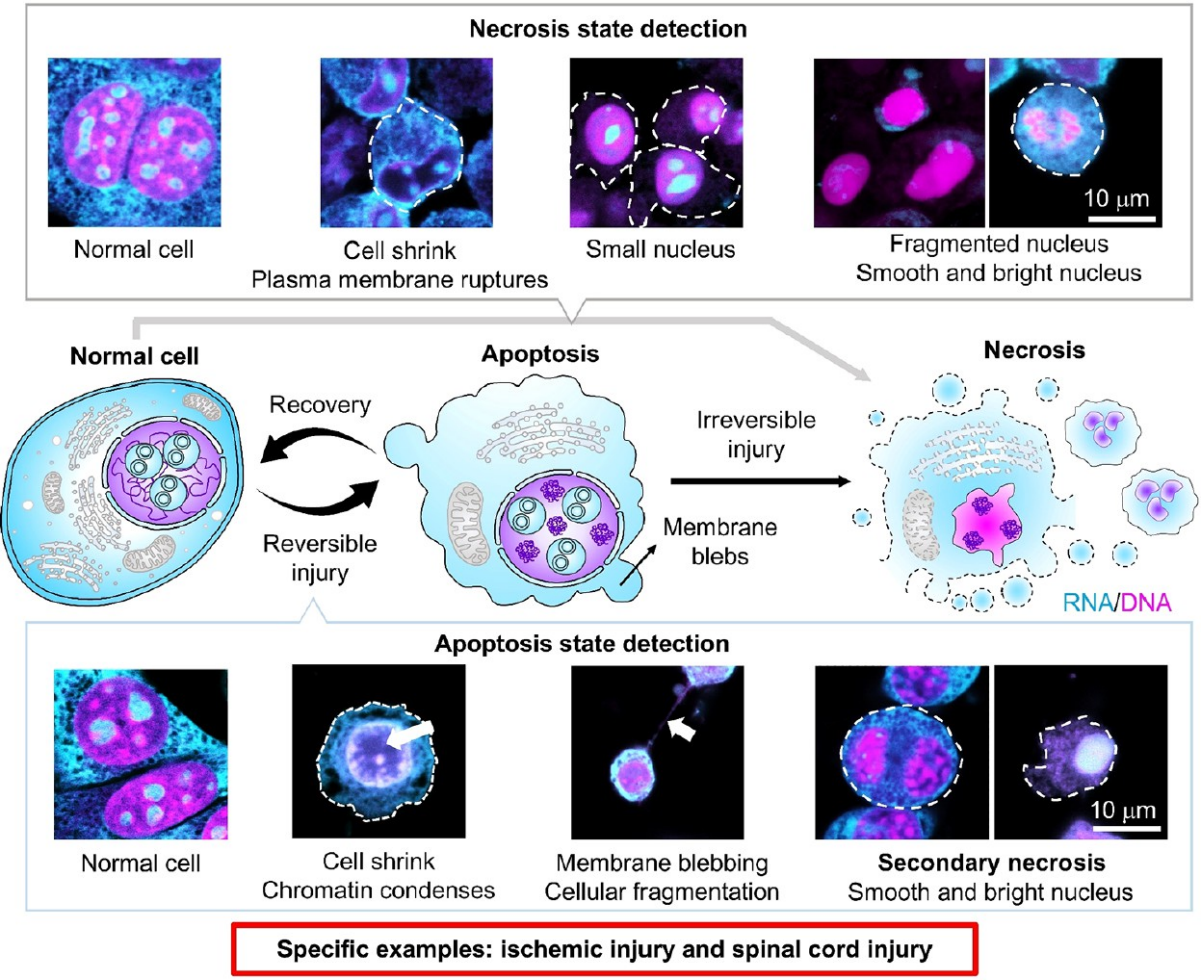
Distinct morphological features from apoptosis to necrosis/necroptosis stages can be distinguished using RNA/DNA staining. Reprinted from ref 98. Copyright 2025, AAAS, licensed under CC BY 4.0.

### Retention Time: A Barrier to Recording a Complete Cellular History

The retention time of a fluorophore in a subcellular organelle directly influences its long-term imaging performance. A probe should remain localized to its target without disturbing cellular metabolic activity while also avoiding nonspecific leakage caused by serum or other extracellular components.^132^ Fluorophore–target interactions can change over time due to probe diffusion, weak binding affinity, target dynamics, or environmental changes. Such time-dependent specificity must be considered and evaluated when designing experiments, especially for live-cell or long-term imaging studies, to ensure that the signal fidelity reflects the target rather than the artifact. Both inadequate retention and prolonged sequestration can compromise signal specificity and biological relevance. By achieving these two important criteria, the complete cellular history can be recorded in a more precise manner (Figure 8).

**Figure 8:**
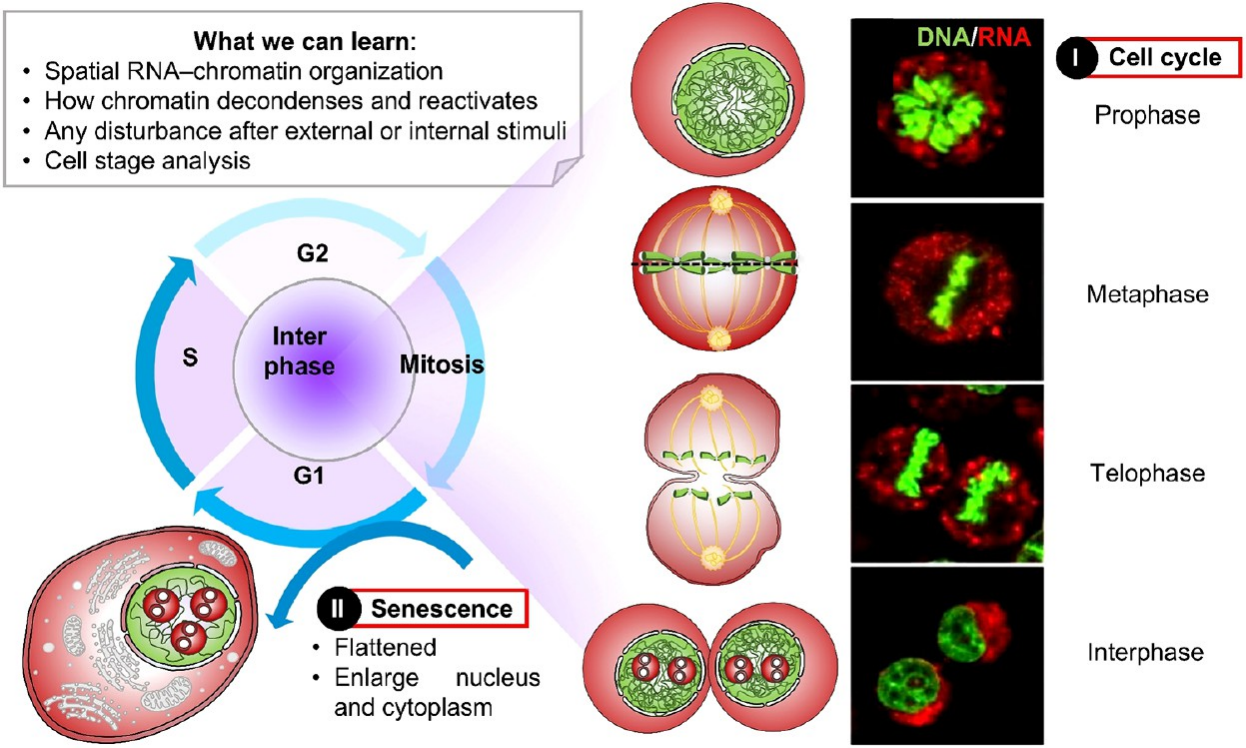
Changes in RNA and DNA distribution serve as markers for identifying the specific phases of the cell cycle. Reprinted with permission from ref 67. Copyright 2019, Wiley-VCH.

### Interference of Other Biomolecules: Impact on Imaging Accuracy

Nonspecific binding to proteins, lipids, or other biomolecules in the cell might lead to off-target fluorescence, quenching, local photothermal effect, or spectral distortion. Optimizing chemical probes to reduce such interactions (*e.g*., with zwitterionic) is crucial for achieving high target selectivity, particularly in quantitative imaging in a crowded bioenvironment.^133,134^

### Imaging Complexity Arising from Synergistic Therapeutic Diagnostic Attempts

Most studies focus on the synergistic use of imaging and therapeutic functions to eliminate and image cancer cells.^79,135^ However, there are several important considerations for such a synergistic approach, such as “What happens to probe detection once cancer cells begin to die? Will the probe still provide accurate quantitative imaging, or will it bind to other biomolecules due to changes in the biological environment? Moreover, how reliable is the quantitative sensing capability during the dynamic treatment process?” Taken together, such functional-purpose probes must be carefully designed to ensure a meaningful interpretation of imaging outcomes.

### Beyond Simple: Considerations for Accurate Selection and Future Development

In this section, we outline several considerations that both biologists and probe developers should consider when designing bioimaging experiments.

### Complexity Limits In-Depth Understanding of How Staining Works

Advancing next-generation probes requires an in-depth understanding of how they interact with their biological targets. Once inside the cells, probes encounter a highly crowded environment composed of biomacromolecules, ions, membranes, and other cellular components. This complexity makes it challenging to identify the precise binding sites and determine how these interactions influence imaging outcomes (Figure 9).^136^ A major barrier in understanding probe staining mechanisms arises from the combination of multiple potential chemical binding sites and the heterogeneous nature of the cellular environment. For instance, although **TEG**_**8**_ **-N14** is one of the most promising probes for RNA/DNA imaging because of its high photostability and near-infrared absorption, its practical application remains limited by its low scalability. Incorporation of nitrogen into the acene core can induce an umpolung of the electronic properties, thereby converting acenes into a class of electron-transporting materials. However, when nitrogen atoms are arranged in the ortho or meta positions, the structural motifs exhibit increased susceptibility to nucleophilic attack, which complicates their purification due to facile deprotonation. Therefore, further development of its chemical design, such as structural modification and incorporation of cell-penetrating peptides, liposomes, or any type of nanocarrier, is required to overcome the problems mentioned above. Hence, a deeper understanding of the binding mechanism is necessary for further optimization. Although docking simulation studies have suggested the possibility of groove binding with DNA and hydrogen bonding with RNA, factors such as multiple binding sites, protonation/deprotonation states, and the type of DNA or RNA may also influence their performance. Therefore, a detailed understanding of its mechanism is necessary before proceeding to further development.^98,124^ Although theoretical calculations are widely used to investigate such mechanisms, they remain insufficient for fully elucidating the staining mechanism.

**Figure 9:**
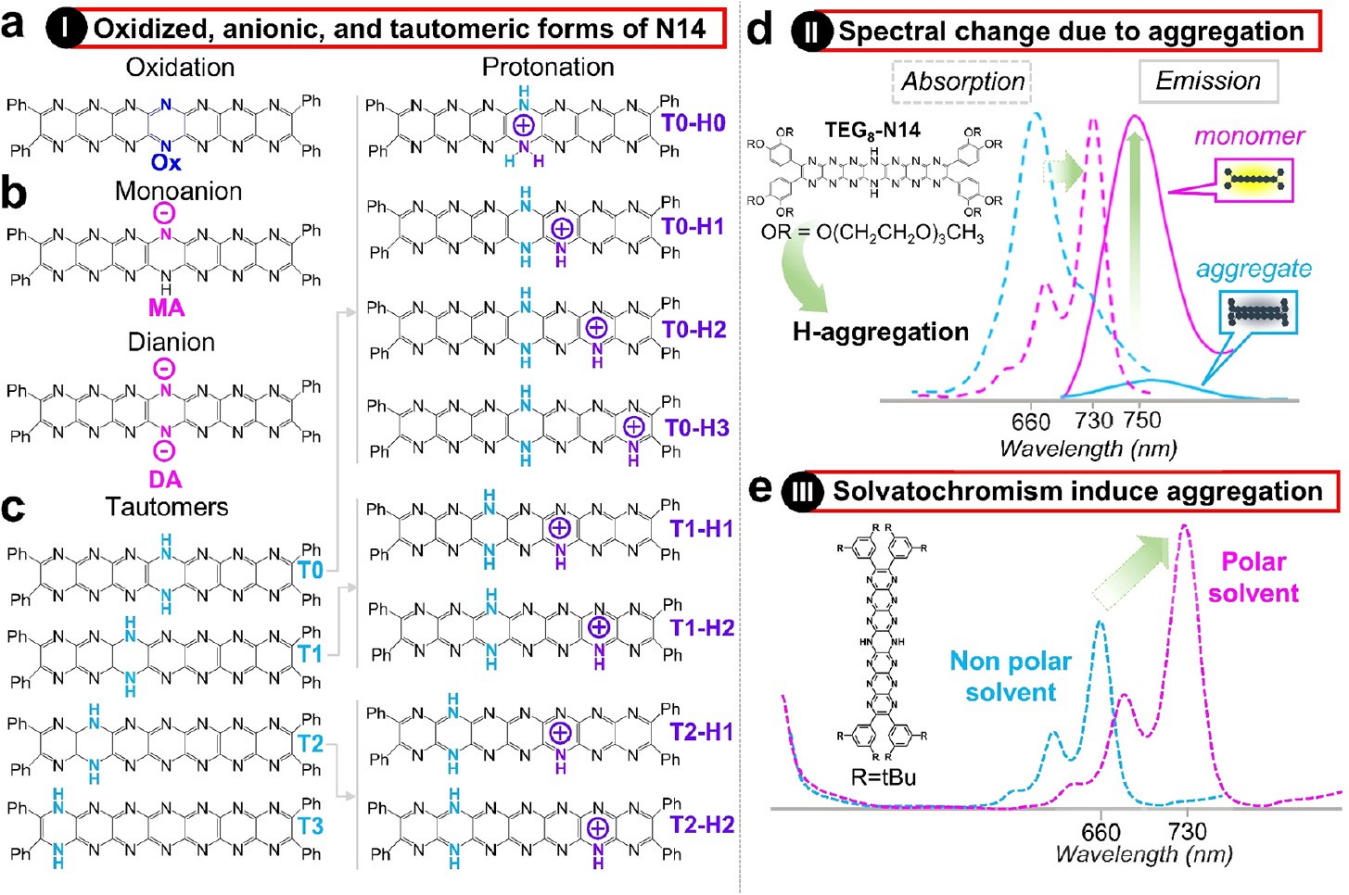
Protonation, redox, aggregation, and solvatochromic responsiveness of the N_14_ framework. (a) Structure of the oxidized product (Ox) of Ph_4_H_2_N_14_HEPT. (b) Chemical structures of monoanion (MA) and dianion (DA). (c) Tautomers of Ph_4_H_2_N_14_HEPT (T0, T1, T2, and T3) accessible through concerted double-proton shifts (other possible tautomers are not considered here). Structures of the selected protonated tautomers of Ph_4_H_2_N_14_HEPT calculated in this study. (d) Spectral changes associated with aggregation states. (e) Spectral variations observed in solvents with different polarities, demonstrating solvatochromic behavior.

Owing to recent developments, artificial intelligence and machine learning have contributed to the prediction of biological responses and customization of molecular recognition for guiding probe design and personalized drug delivery.^137^ Despite these advances, the lack of high-quality and standardized AI data sets often leads to fragmented data, inconsistent reports, and a lack of coherence between surface chemistry and biological results. Such facts, in turn, make data interpretation difficult and ultimately create hurdles for validation and reproducibility.

One of the most reliable methods to reveal such a staining mechanism is direct molecular observation and in situ characterization of different chemical structures. As long-term development goals, the following two vital areas are recommended:

#### Scanning Tunneling Microscopy/Atomic Force Microscopy (STM/AFM)

Imaging DNA and RNA with fluorescent molecules at atomic resolution is extremely challenging, but surface techniques such as STM/AFM may offer a possible path toward achieving this goal. STM/AFM are important tools in on-surface chemistry, especially for resolving carbon-based nanostructures synthesized on solid substrates.^138–141^ In earlier studies, STM/AFM enabled the characterization of DNA at the solid–liquid interface; however, the resolution was limited, making it difficult to achieve high resolution.^142,143^ Drying the deposited solution allows the sample to be measured under ultrahigh vacuum, which helps improve the resolution.^144,145^ More advanced ultrahigh-vacuum deposition techniques for macromolecules are still needed. Fortunately, recent progress in electrospray methods has allowed direct deposition onto metal substrates under ultrahigh-vacuum conditions, enabling high-resolution STM/AFM imaging of species such as proteins,^146^ peptides,^147^ glycans,^148^ and DNA molecules.^149^ Even glycans bonded to proteins and lipids can be observed and studied by STM at the single-molecule level.^150^ Similarly, DNA or RNA together with fluorescent molecules can be deposited onto a surface using electrospray techniques and subsequently investigated by STM/AFM under ultrahigh-vacuum conditions. In addition, we propose an improved approach: first, DNA/RNA is deposited onto the substrate by electrospray, and then fluorescent molecules are deposited onto the partially DNA/RNA-covered surface using organic molecular beam epitaxy (OMBE), allowing them to bind directly to the DNA/RNA on the substrate. Next, the binding sites between DNA/RNA and fluorescent molecules can be visualized by using STM/AFM characterization. This information on the binding sites is very important as it reveals how the interactions occur and the types of interactions that take place. Moreover, scanning tunneling spectroscopy (STS) can detect the highest occupied molecular orbital (HOMO) and the unoccupied molecular orbital (LUMO) of fluorescent molecules.^151^ When these molecules bind to DNA/RNA, their electronic states shift, leading to variations in the HOMO–LUMO gap, which indicates the binding mode and interaction strength. STS enables the direct probing of these changes, offering a clearer picture of how fluorescent molecules interact with DNA/RNA and the strength of these interactions. For example, utilizing a CO-terminated tip for NC-AFM enables the imaging of ssDNA and the corresponding self-assembled structures with subnanometer resolution (Figure 10a). This high-resolution technique clearly reveals the folded conformation and carbon backbone structure of dehydrated ssDNA oligomers on the Au(111) surface (Figure 10b,c). The acquired experimental images show excellent agreement with the structures obtained from the molecular dynamics simulations (Figure 10d,e), confirming the observed structural details. This approach provides a powerful pathway for the atomic-scale characterization of single biomolecular strands.

**Figure 10:**
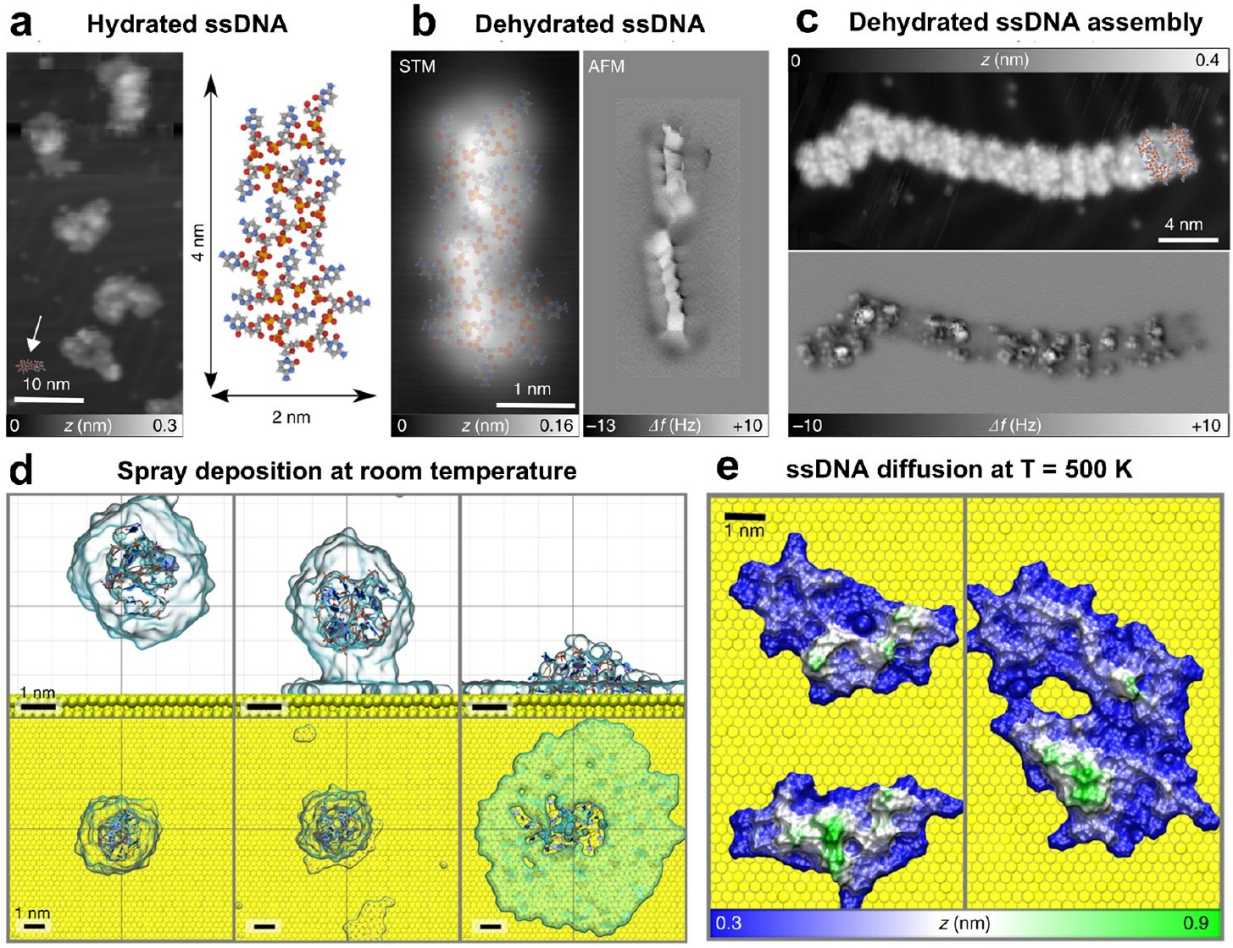
Atomic-scale characterization of ssDNA and molecular dynamics simulations. (a) STM image of hydrated ssDNA after spray deposition at room temperature. The right side shows a representative ssDNA structure on Au (111) obtained from molecular dynamics (MD) simulations. (b) STM image of a dehydrated single 20-cytosine ssDNA oligomer after annealing at 440 K, and the corresponding high-resolution constant-height AFM image, both acquired with a CO-terminated tip. (c) STM image of self-assembled dehydrated ssDNA oligomers after heating to 500 K, and the corresponding AFM image. (d) MD simulation side and top views depicting the adsorption of a water droplet containing one ssDNA strand onto Au(111) (water is shown as a transparent surface). (e) 500 ns MD simulation at 500 K capturing the diffusion-driven assembly of two strands via intermolecular interactions. Reprinted from ref 149. Copyright 2019, Springer Nature; licensed under CC BY 4.0.

#### In Situ Characterization

To better understand the underlying staining mechanisms of molecular probes, it is necessary to compare the different chemical structures of the probes and evaluate their performance in biological environments. Different chemical structures, such as alkyl chains, donor–acceptor motifs, and other functional groups, can affect the binding modes, photophysical properties, cell permeability, and interactions with biomolecules. Therefore, in-depth investigations using techniques such as spectroscopic measurements, isothermal circular dichroism, microscale thermophoresis, or X-ray crystallography are required to clarify the structure–function relationships that influence probe selectivity, brightness, and cellular behavior (Table 1). These insights help improve probe selectivity, brightness, and behavior in cells and are essential for designing more reliable and effective probes for imaging and other biological applications.

**Table 1:** Techniques Used to Study Probe–DNA Binding with Site-Specific Interactions

technique	example application
circular dichroism (CD) spectroscopy	monitors conformational changes in the DNA structure and identification of groove binding or intercalation
microscale thermophoresis (MST)	measurement of direct binding affinity (*K*_d_) of a molecule to DNA to reveal sequence preference, like preferential binding complexes for GC-rich vs AT-rich sequences
thermal melting (Tm) experiments	detects changes in DNA duplex stability in the presence of a drug, such as the evaluation of sequence-specific thermal stabilization
X-ray crystallography	enables atomic-level resolution of compound–DNA interactions, such as structural elucidation of binding modes like intercalation with sequence-specific preferences by complexes relative to groove binding
in-liquid AFM	enables visualization of DNA topologies in hydrated form, such as detection of DNA triplex formation at base-pair resolution

### Rethinking the Use of AI for Data Analysis with Attention to Ethical Concerns

Analyzing large and complex imaging data sets is time-consuming and requires high-level microscopy expertise. AI-based platforms, such as Leica’s Aivia and Imaris, have been developed to automate segmentation and quantification. Although these platforms offer many benefits, they must be applied with caution. Several limitations, such as insufficient training data, variable data quality, and algorithmic biases, can affect the data reliability and raise ethical concerns. Establishing guidelines for the responsible use of AI in biological imaging and performing deep learning with professionals is critical to ensure scientific validity, reproducibility, and transparency.^152,153^

### Solid Biological Knowledge Is a Prerequisite for Accurate Results

Biological systems are highly dynamic and interconnected, and even small differences in cellular states or environments can significantly affect experimental results. Accurate interpretation of imaging data, therefore, requires a deep understanding of biological principles and context. Without this foundation and high standardization, there is a risk of misattributing artifacts, overlooking context-dependent phenomena, or drawing ambiguous conclusions, which can obscure the true underlying processes. Therefore, standardized protocols are needed to minimize linkage errors.

### Careful Selection of Each Experimental Component for Characterization

Characterization is essential before advancing to the next stage of probe development. Below, we outline several important considerations for probe characterization.

#### Sensitivity of Detection Equipment

The performance of each probe is determined by the characterization of its photophysical properties after binding to the cells. Key components, such as detectors, lasers, objective numerical apertures, and other optical elements, must be properly calibrated to maintain stable signals and reduce noise during the experiments. Inadequate calibration and the lack of a positive control can lead to false positives and mislead the interpretation of the probe behavior (Figure 11).

**Figure 11:**
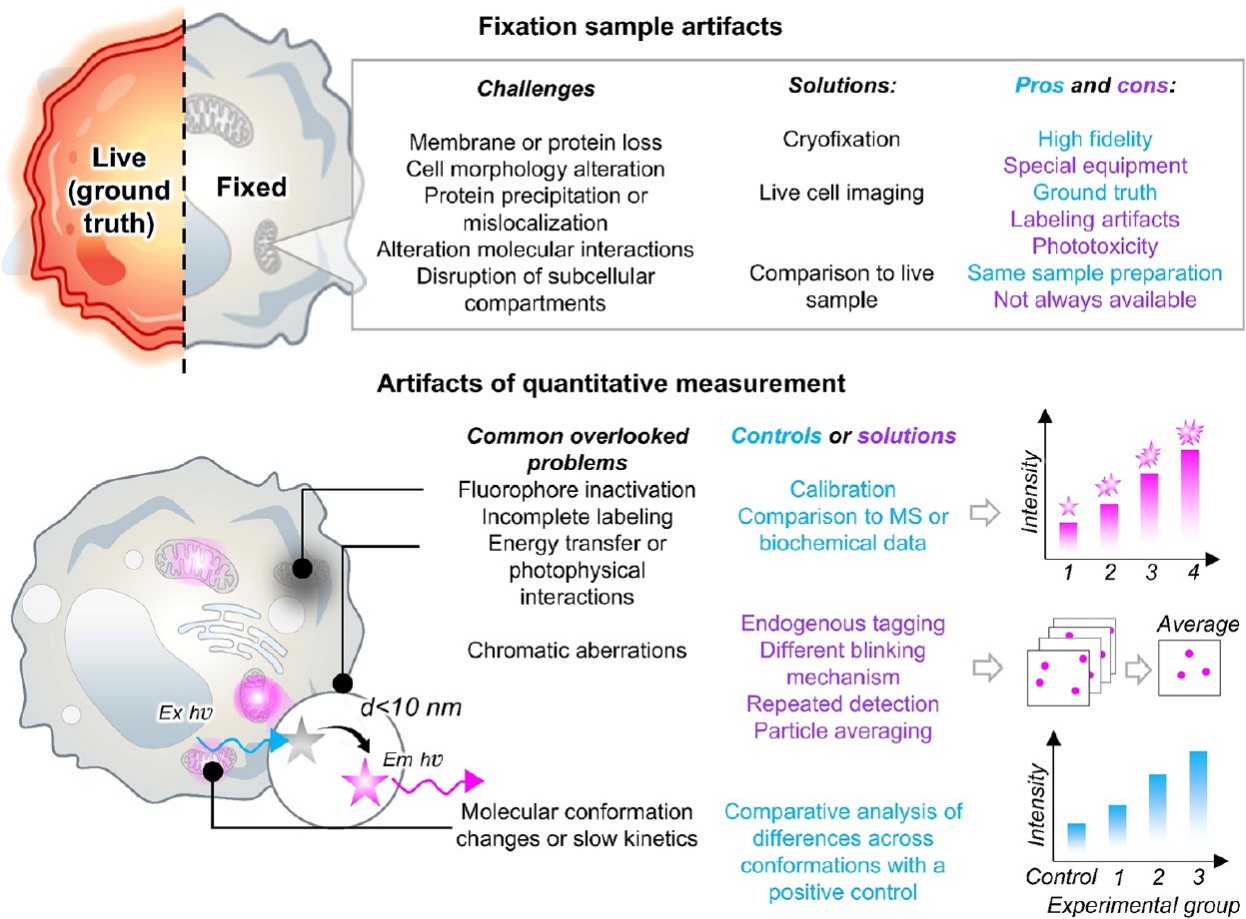
Overlooked problems in fluorescence imaging and proposed solutions for improving its accuracy.

#### Fixation Selection and Why There Are No One-Size-Fits-All Solutions

At the initial stage of sample labeling, fixation should preserve the structural integrity of the specimen that closely reflects the state of the living sample; however, this process is susceptible to the introduction of artifacts. There is no universal fixation protocol, as a fixative that performs well under one set of conditions may be suboptimal under another.^65^ For example, 4% paraformaldehyde (PFA) is widely used because it provides good preservation of morphology; however, it penetrates samples relatively slowly. Penetration can be improved by the addition of 10% ethanol, although this may alter organelle morphology. Cold methanol fixation is commonly employed for certain cytoskeletal structures but fails to preserve cellular membranes. Glutaraldehyde offers superior structural preservation, yet it can modify epitopes, reduce immunogenicity, and impair the performance of affinity-based labels. Membrane permeabilization, while frequently required, inevitably extracts lipids and disrupts cellular membranes and organelles. To circumvent the need for permeabilization, smaller or membrane-permeant affinity-based probes can be employed. Finally, incomplete fixation may leave some biomolecules moving, leading to apparent protein localization that differs from their native state. Therefore, testing multiple fixation strategies is generally recommended for each target structure or model system.

#### Cell Culture Platform

The most important consideration when choosing a cell culture platform for imaging is its ability to closely mimic the native tissue environment. Factors such as cell type, extracellular matrix composition, and culture platform can significantly influence the probe uptake, distribution, and biological responses. Another critical requirement when performing cell culture for imaging is the imaging duration.^112,125^ For long-term imaging, a 3D environment may be necessary to prevent alterations, such as nuclear flattening. For short-term tracking, factors such as nonspecific binding and the retention time of the probe to bind to specific subcellular organelles must be carefully evaluated.

### Commercialization: Balancing Scalability, Quality Control, Safety, and Cost

Translating imaging probes from the laboratory to commercial use requires a balance between scalability, quality control, safety, and cost. Commercial fluorophores must meet standards for consistency while remaining broadly accessible to both academic and industrial researchers. Ensuring this balance is essential for effective application in biological research. Achieving this balance is critical for the broad practical application of whole RNA/DNA imaging (Table 2).

**Table 2:** Representative Application of Simultaneous Imaging of the Nucleoli, Cytoplasm, and Nucleus^a^

probe	observation condition (λ_ex_/λ_em_, nm)	model system	application	refs
**AO**	457/525 (DNA)	MO3 cell	oligodendrocyte injury detection	^87^
	457/625–645 (RNA)	MEF and NIH-3T3 cells	apoptosis, necrosis, and necroptosis detection	
		Thy1-YFP mouse optic nerve	chemical ischemia detection	
		mouse spinal cord	spinal cord injury detection	
	488/530 (DNA)488/650 (RNA)	lens epithelial cell	galactosemia detection	^154^
	488/530 (DNA)488/640 (RNA)	histopathological staining of primary renal cell carcinoma (RCC)	primary renal cell carcinoma (RCC) detection	^155^
	467/520 (DNA) X-ray (radiotherapy)	human musculoskeletal sarcoma	photodynamic or radiotherapy for human musculoskeletal sarcomas	^156^
	N.A.	zebrafish	oogenesis stage detection	^157^
	490/510–560 (DNA)490/600–650 (lysosomes)	mixed glial cell culture (astrocyte/microglia)	discriminatory marker of microglia in different types of astrocytes	^158^
	488/580–630 (RNA)	duffy positive and duffy knockout miceperipheral blood of anemic mice	identification of infected erythrocytes and reticulocytes in malariadetermine the maturity of reticulocytes	^159^
	N.A.	chemo-sensitive mouse osteosarcoma cell	photodynamic therapy of multidrug resistance (MDR) of mouse osteosarcoma	^160^
	N.A.	bone marrow cell	multiple myeloma detection	^161^
		spermatozoa	genome damage in reticulocyte detection	^162^
	N.A.	keratinocytes	classification of resting, proliferating, and differentiating cells	^163^
**carbon quantum dots**	488/500–560(DNA) 543/570–650 (RNA)	*Caenorhabditis elegans* (*C. elegans*) cell division	time-lapse imaging of chromatin and nucleoli during cell division and *C. elegans* growth	^67^
**DR1**	560/600–650 (DNA)640/650–700 (RNA)	HeLa cell	high-content screening platform (cell cycle)	^97^
**TEG**_**8**_ **-N14**	640/650–720 (RNA) 730/740–850 (DNA)	NIH-3T3 cell	necrosis detection (live)discrimination of apoptosis, necrosis, and necroptosis (fix)	^98^
		ARPE-19 cell	senescence at low and high passage	

a N.A.: not available.

## Prospective and Proposal

This review focuses on RNA/DNA imaging, with the aim of developing practical molecular nanoarchitectonics. Although this objective is fundamental, we examine whether useful technologies have been developed from the perspective of the user and discuss the necessary future directions (Figure 12). It is essential to develop molecular materials and techniques that can distinguish between DNA and RNA, image them accurately in real time, and closely monitor their behavior. Therefore, there is an urgent need for a single-step, multiplexed RNA–DNA imaging technique that works under conditions compatible with both RNA and DNA. Integrating both DNA and RNA information enables researchers to better understand how structural genomic characteristics affect transcriptional heterogeneity across different cell types and conditions while minimizing artifacts. Among these methods, fluorescent imaging using chemical probes is the most convenient for labeling RNA and DNA, as it can be performed without specialized biological knowledge.

**Figure 12:**
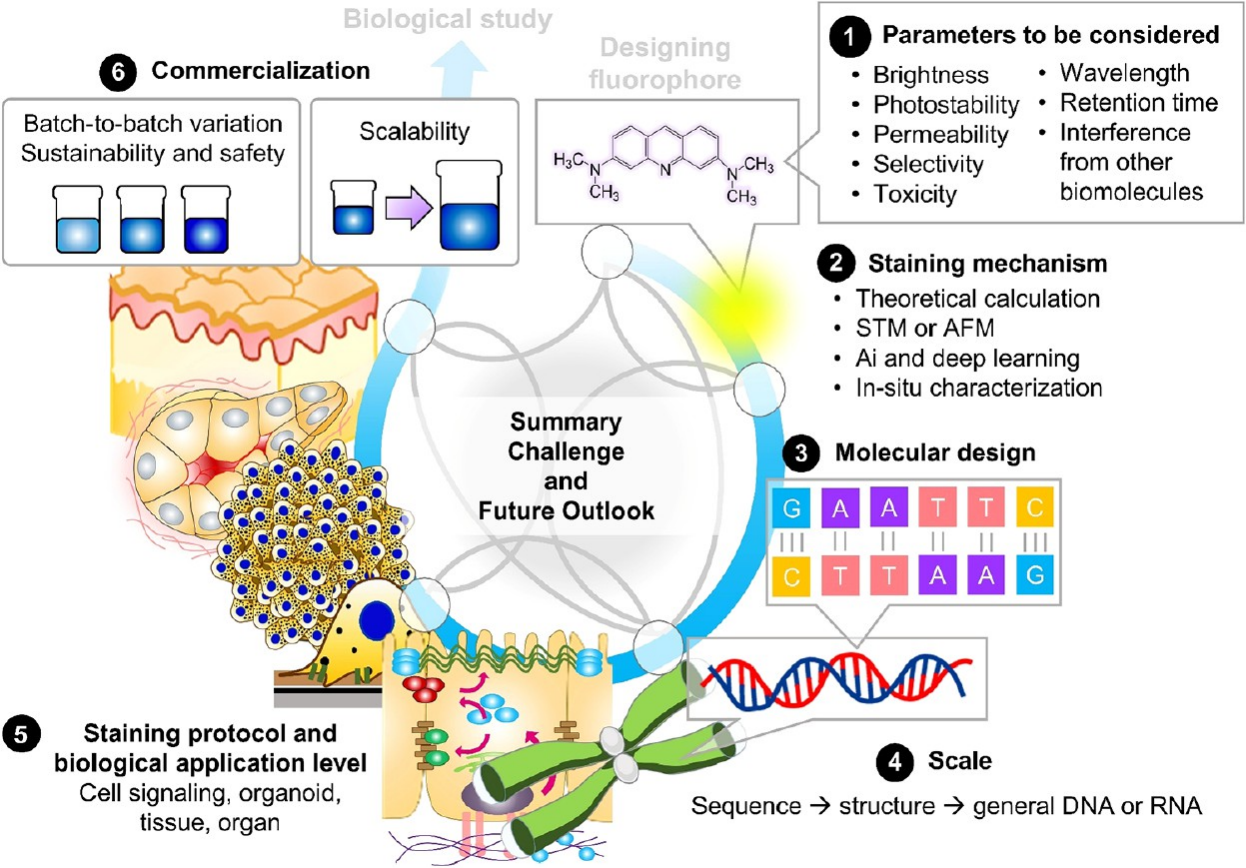
Proposed development roadmap for commercial fluorophores based on the mechanism study and applied biological research.

Although perfect probes are rare, certain key design elements are essential for their optimal performance. It is crucial to carefully select fluorescence excitation and emission wavelengths that are tailored to the application. The brightness of the probe after intracellular uptake is also important. Probes with appropriate sensitivity are far more preferable to overly sensitive ones. For live-cell imaging, careful consideration must be given to the addition of cell-permeable substances. Multiplexed fluorescence imaging enables the simultaneous detection of multiple intracellular targets and reveals intertarget relationships in a single step. In long-term imaging studies, time-dependent specificity must be carefully considered and evaluated. This is particularly important for achieving high target selectivity in the quantitative imaging of crowded biological environments.

Recently, probes that meet many of these criteria have been developed. For instance, **TEG**_**8**_ **-N14**, which is discussed in this review, demonstrated the importance of N-rich structural compounds in enabling the simultaneous visualization of RNA and DNA using dual near-infrared excitation. This breakthrough not only minimizes phototoxicity and spectral crosstalk due to NIR excitation but also demonstrates broad applicability in measuring multiple types of cellular damage in different cell types. Dual-functional probes are essential for advancing diagnostics and therapeutics. To improve their functionality, strategies should focus on modifying their chemical structures to enhance cell permeability, brightness, and performance.

This review aims to provide an overview of the latest developments in bioimaging, highlight currently overlooked intracellular imaging issues, and offer biological insights to encourage researchers in various fields to explore new strategies related to their research. Our goal is to help biologists select the most appropriate approach for their research and to support future researchers in developing next-generation RNA/DNA probes, from conceptual ideas to commercialization, from a biologist’s perspective. We hope to provide a useful guide. Below, we summarize the requirements for future RNA/DNA probe molecules.

### Ideal Criteria for Future RNA/DNA Probes

(1) High sequence specificity— able to recognize one exact DNA or RNA sequence
(2) NIR excitation and emission—avoid autofluorescence and light scattering; low spectral overlap with commonly used probes
(3) Minimal light-induced chemical reactions—low photothermal effect and phototoxicity to avoid forming reactive byproducts under illumination. Such properties allow repeated imaging of both slow and fast biological processes
(4) Single-parameter sensitivity—for example, responds only to a change in nucleic acid, not pH, aggregation, stable performance, despite salt fluctuations or other environmental factors
(5) High accumulation at the specific intracellular target—achieve a high signal-to-noise ratio to ensure compatibility with any microscope, performs well even in crowded intracellular spaces
(6) A stable signal over time—maintains sufficient signal at the target during long experiments
(7) High cell permeability—improves brightness and overall imaging performance
(8) Low cross-reactivity with other probes—allows reliable costaining
(9) Small size—facilitates easy diffusion to tightly connected cells.
(10) High photostability—compatible with super-resolution microscopy, including STED, which uses intense laser power
(11) Minimal perturbation— does not interfere with normal cellular processes or gene expression, minimal interaction with membranes to avoid sticking to lipid bilayers
(12) Good performance across cell types— broad applicability to various types of mammalian cells, and if possible, for plants, bacteria, and model organisms
(13) Minimal precipitation—maintains solubility during preparation
(14) Minimal-wash ability—minimizes any perturbance
(15) High chemical stability—maintains its performance over time in long-term storage conditions
(16) Compatibility with flow cytometry—adaptable to various imaging platforms
(17) Choose the correct fixation method— imaging outcomes must not be affected by the fixation process
(18) Easy functionalization— can be linked to targeting ligands, peptides, or antibodies
(19) Long shelf life—stable during long-term storage
(20) Scalable and cost-efficient synthesis—feasible for large-scale studies
(21) High reproducibility across batches—consistent performance

Of course, ensuring reliable quantitative sensing capabilities during dynamic therapeutic processes requires solid biological knowledge and accurate results. Other important factors include the careful selection of each experimental component for characterization and the provision of a 3D environment for long-term imaging in cell culture platforms. It is also essential to balance scalability, quality control, safety, and cost for commercializing these technologies. A comprehensive evaluation using in situ characterization techniques is also essential. Novel approaches, such as the introduction of AI technology and direct molecular-level observation using probe microscopy, are expected to make a significant contribution. Nanoarchitectonics for molecular design and refinement of evaluation methods will lead to the development of multiplexed RNA-DNA imaging that functions in a single step under conditions compatible with both RNA and DNA. This will make it more useful for users.

## Vocabulary

Autofluorescence: is defined as the natural emission of light by endogenous fluorophores in biological systems when they absorb light
Absorbance coefficient: is a quantitative measurement that indicates how strongly a molecule absorbs light
Phototoxicity: refers to cellular damage caused by light exposure that may alter the normal cellular behavior
Apoptosis: a programmed form of cell death that causes changes in the cell morphology while maintaining membrane integrity
Necrosis: an uncontrolled form of cell death that shows loss of membrane integrity and release of intracellular contents
